# Supplementary material for: A detailed kinetic model of Eastern equine encephalitis virus replication in a susceptible host cell
Source: bioRxiv. 2024 Dec 26:2024.12.13.628424. Preprint. [Version 2] doi: 10.1101/2024.12.13.628424 (PMC11703215; doi:10.1101/2024.12.13.628424)
Supplement: 1 [file NIHPP2024.12.13.628424V2-supplement-1.pdf]

bioRxiv preprint doi: https://doi.org/10.1101/2024.12.13.628424; this version posted December 26, 2024. The copyright holder for this preprint (which was not certified by peer review) is the author/funder, who has granted bioRxiv a license to display the preprint in perpetuity. It is made available under aCC-BY 4.0 International license.

Supporting Information

S1 Table. Mechanistic model description including species, parameters, and rate rules.

S2 Table. Key resources table.

bioRxiv preprint doi: https://doi.org/10.1101/2024.12.13.628424; this version posted December 26, 2024. The copyright holder for this preprint (which was not certified by peer review) is the author/funder, who has granted bioRxiv a license to display the preprint in perpetuity. It is made available under aCC-BY 4.0 International license.

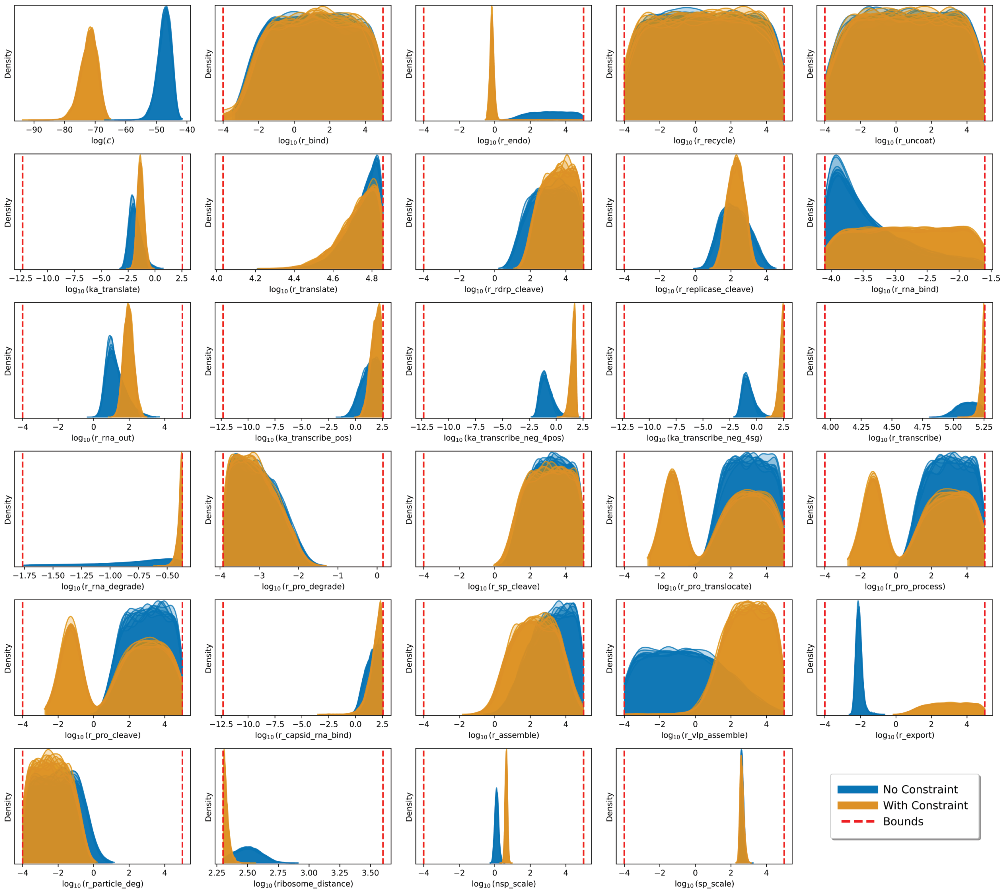

S1 Fig. Representative 1D marginal distributions for estimated parameters after calibrating the model with experimental data with and without the RNA genome strand ratio constraint. 26 of the 36 model parameters in the BioNetGen model are calibrated as well as 2 scaling factors, `nsp_scale` and `sp_scale`, which are used to scale nonstructural and structural polyprotein concentrations to luminescence. These distributions are from the posterior ensemble of 2,500 parameter sets after calibrating the model given different set of initial particles. Specifically, the model was calibrated 10 times with the RNA genome strand constraint (blue) and 10 times without the constraint (orange). Vertical, dashed red lines indicate the parameter bounds provided during model calibration.

bioRxiv preprint doi: https://doi.org/10.1101/2024.12.13.628424; this version posted December 26, 2024. The copyright holder for this preprint (which was not certified by peer review) is the author/funder, who has granted bioRxiv a license to display the preprint in perpetuity. It is made available under aCC-BY 4.0 International license.

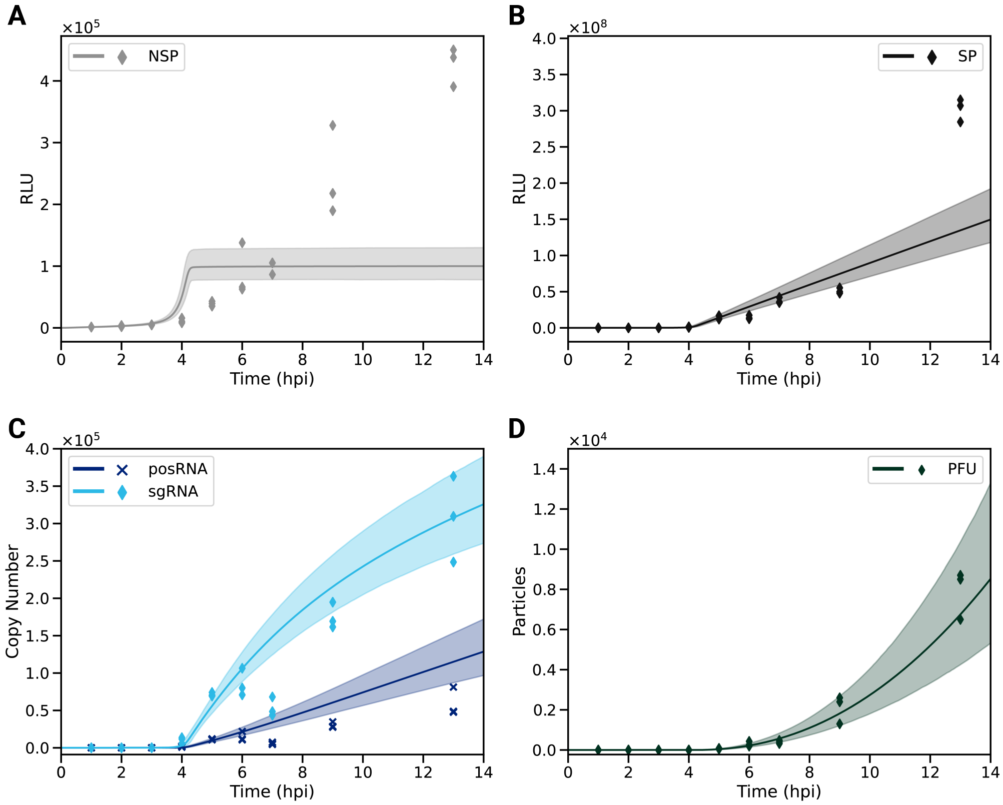

S2 Fig. The model fits to the experimental data in linear space when performing parameter estimation with the RNA genome strand ratio constraint. The posterior samples from model calibration (n = 2,500) trajectory weighted means (line) and 95th percentiles (shaded) are shown in all panels. The mean and standard deviation of experimental data ($n = 3$ replicates) are plotted from the protein luminescence assays, RT-qPCR, and plaque assay (see Materials and methods). Fits to protein luminescence data of nonstructural protein 3 (nsP) and structural protein (sP) in relative light units (RLUs) are shown in panels (A) and (B) respectively. (C) RNA dynamics of the positive-sense genome (posRNA, dark blue) and subgenomic RNA (sgRNA, light blue) are shown. (D) Model trajectories of viral particle production of plaque-forming, infectious particles (PFU).

bioRxiv preprint doi: https://doi.org/10.1101/2024.12.13.628424; this version posted December 26, 2024. The copyright holder for this preprint (which was not certified by peer review) is the author/funder, who has granted bioRxiv a license to display the preprint in perpetuity. It is made available under aCC-BY 4.0 International license.

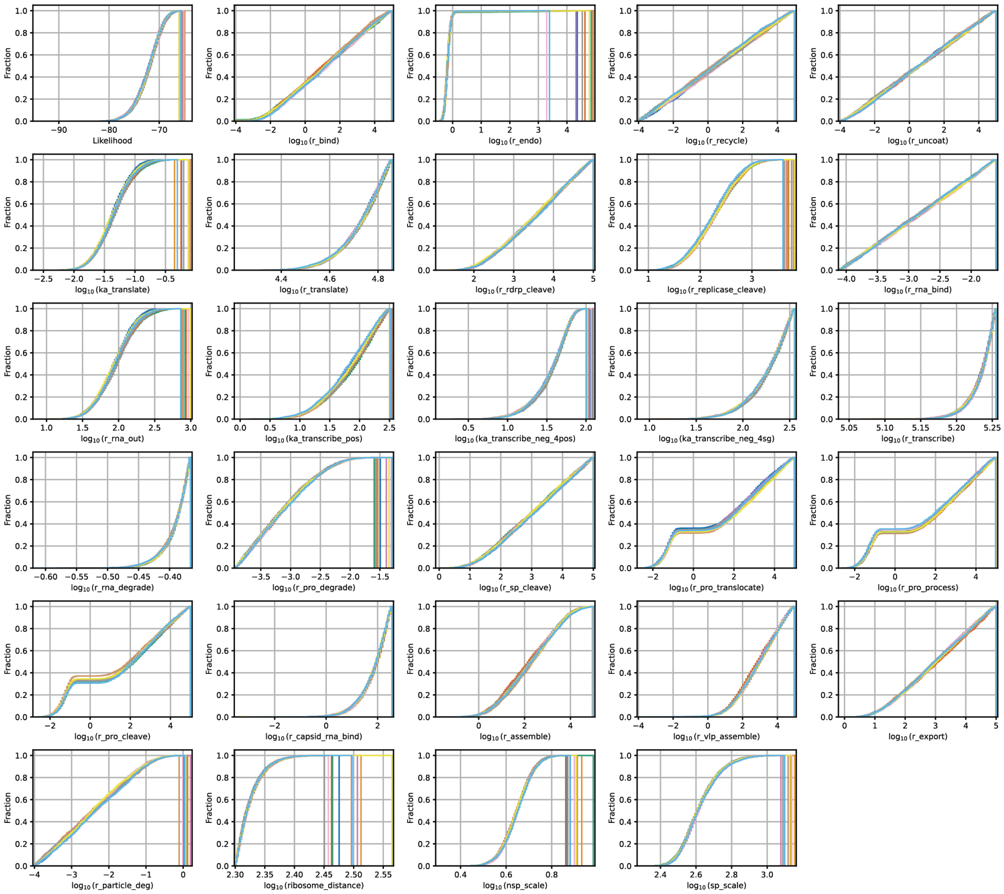

S3 Fig. The marginal posterior cumulative distributions of the estimated parameters. Model calibration to the RNA genome strand ratio constraint and experimental data was run 10 times given different initial configurations.

bioRxiv preprint doi: https://doi.org/10.1101/2024.12.13.628424; this version posted December 26, 2024. The copyright holder for this preprint (which was not certified by peer review) is the author/funder, who has granted bioRxiv a license to display the preprint in perpetuity. It is made available under aCC-BY 4.0 International license.

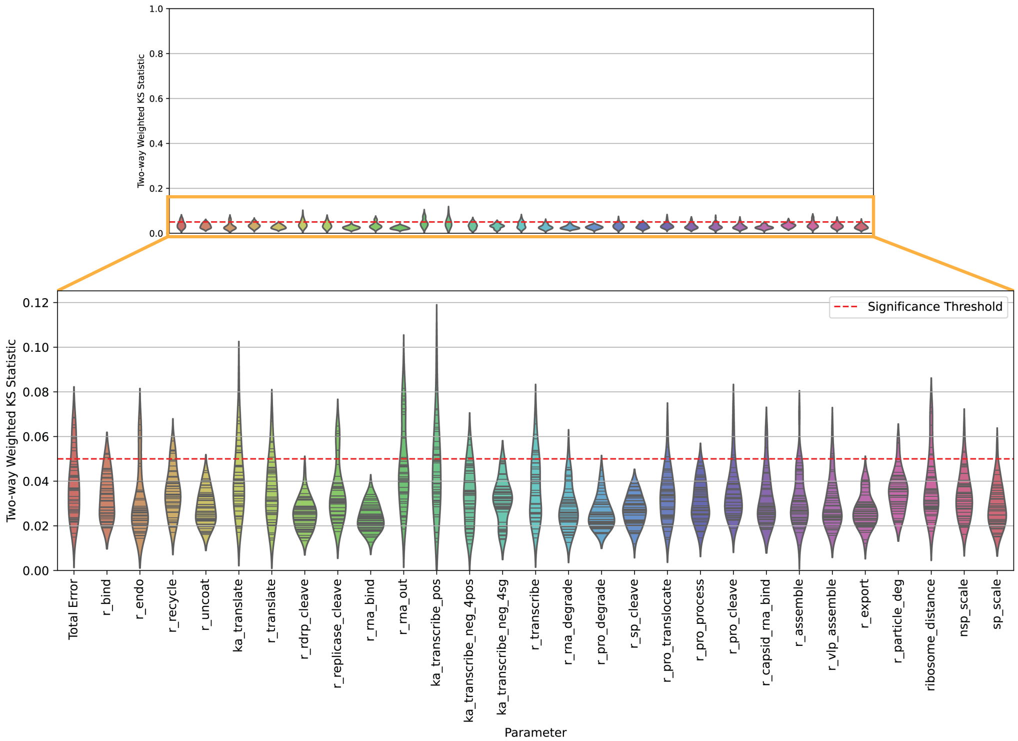

S4 Fig. Pairwise Komolgorov-Smirnov statistic distributions for all model parameters across 10 independent runs of model calibration. Using the resulting marginal cumulative distributions generated by each run of model calibration, as shown in Fig S3, the two-way weighted Komolgorov-Smirnov statistic between each combination of runs (10 runs, 45 pairs for comparison) for the likelihood (see Eqn 10) and 28 calibrated parameters. The black lines in each violin plot represent individual KS statistic for a pair of runs.

bioRxiv preprint doi: https://doi.org/10.1101/2024.12.13.628424; this version posted December 26, 2024. The copyright holder for this preprint (which was not certified by peer review) is the author/funder, who has granted bioRxiv a license to display the preprint in perpetuity. It is made available under aCC-BY 4.0 International license.

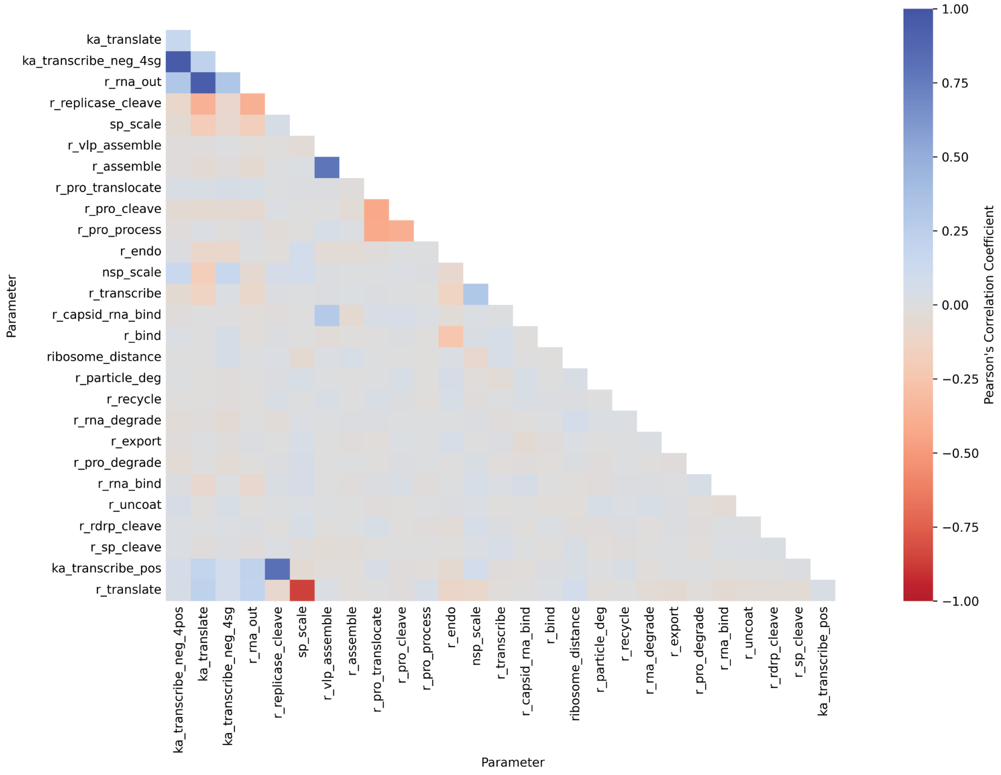

S5 Fig. Pairwise calibrated parameter correlations. The Pearson correlation coefficients between calibrated parameters ($n = 28$, 26 model parameters and 2 scaling factors) are calculated from the parameter set ensemble ($n = 2,500$).

bioRxiv preprint doi: https://doi.org/10.1101/2024.12.13.628424; this version posted December 26, 2024. The copyright holder for this preprint (which was not certified by peer review) is the author/funder, who has granted bioRxiv a license to display the preprint in perpetuity. It is made available under aCC-BY 4.0 International license.

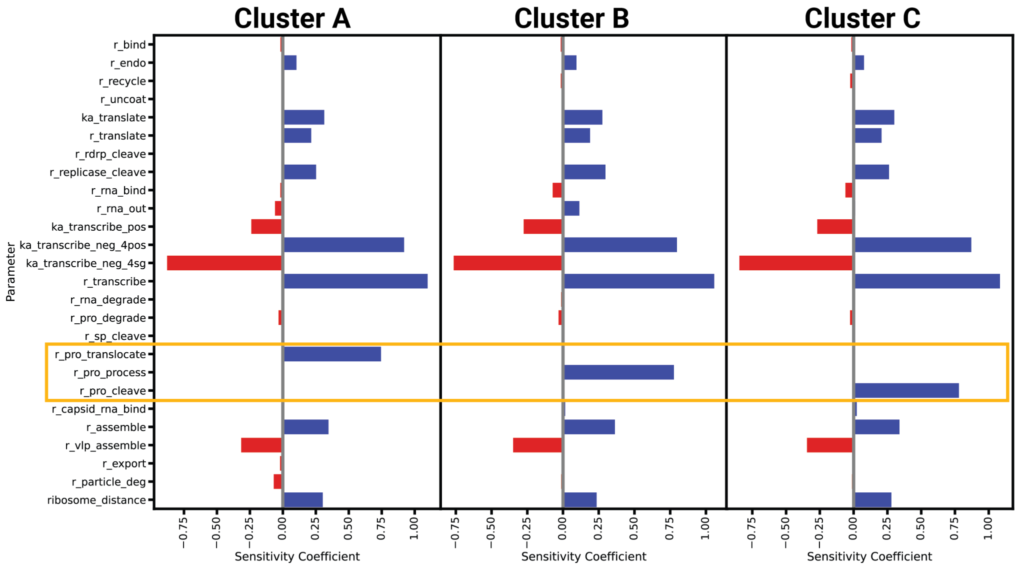

S6 Fig. Cluster-based linear regression sensitivity analysis reveals three modes of post-translational processing dynamics. The sensitivity coefficients when sampling around the best posterior parameter set from each individual cluster $(n = 3)$ using the same method described in Fig 7A. As shown in Fig 6, the left plot corresponds cluster in which `r_pro_translocate` is the rate-limiting parameter. The center shows the resulting sensitivity coefficients when sampling parameters around the best sample cluster in which `r_pro_process` is rate-limiting. The right plot corresponding to the sensitivity coefficients for model parameters using the best parameter set from the cluster in which `r_pro_cleave` is rate-limiting.

S1 File. The mechanistic model in the BioNetGen Language format.

S2 File. The mechanistic model in the Systems Biology Markup Language format.
